# Mobile health for non-communicable diseases in Sub-Saharan Africa: a systematic review of the literature and strategic framework for research

**DOI:** 10.1186/1744-8603-10-49

**Published:** 2014-06-13

**Authors:** Gerald S Bloomfield, Rajesh Vedanthan, Lavanya Vasudevan, Anne Kithei, Martin Were, Eric J Velazquez

**Affiliations:** 1 Department of Medicine and Duke Clinical Research Institute, Duke University, 2400 Pratt Street, Durham, North Carolina 27705, USA; 2 Duke Global Health Institute, Trent Drive, Durham, North Carolina 27705, USA; 3 Zena and Michael A. Wiener Cardiovascular Institute, Icahn School of Medicine at Mount Sinai, One Gustave L. Levy Place, Box 1030, New York City, NY 10029, USA; 4 Department of International Health, Johns Hopkins Bloomberg School of Public Health, Baltimore, Maryland, USA; 5 School of Medicine, College of Health Sciences, Moi University, Nandi Road, Box 2600–30100, Eldoret, Kenya; 6 The Regenstrief Institute, Inc. and Department of Medicine, Indiana University School of Medicine, 410 West 10th Street, Suite 2000, Indianapolis, Indiana 46202, USA; 7 USAID-Academic Model Providing Access to Healthcare (AMPATH) Partnership, PO Box 4606, Nandi Road, Eldoret, Kenya

**Keywords:** Mobile health, Non-communicable disease, Sub-Saharan Africa, Systematic review

## Abstract

**Background:**

Mobile health (mHealth) approaches for non-communicable disease (NCD) care seem particularly applicable to sub-Saharan Africa given the penetration of mobile phones in the region. The evidence to support its implementation has not been critically reviewed.

**Methods:**

We systematically searched PubMed, Embase, Web of Science, Cochrane Central Register of Clinical Trials, a number of other databases, and grey literature for studies reported between 1992 and 2012 published in English or with an English abstract available. We extracted data using a standard form in accordance with Preferred Reporting Items for Systematic Reviews and Meta-Analyses guidelines.

**Results:**

Our search yielded 475 citations of which eleven were reviewed in full after applying exclusion criteria. Five of those studies met the inclusion criteria of using a mobile phone for non-communicable disease care in sub-Saharan Africa. Most studies lacked comparator arms, clinical endpoints, or were of short duration. mHealth for NCDs in sub-Saharan Africa appears feasible for follow-up and retention of patients, can support peer support networks, and uses a variety of mHealth modalities. Whether mHealth is associated with any adverse effect has not been systematically studied. Only a small number of mHealth strategies for NCDs have been studied in sub-Saharan Africa.

**Conclusions:**

There is insufficient evidence to support the effectiveness of mHealth for NCD care in sub-Saharan Africa. We present a framework for cataloging evidence on mHealth strategies that incorporates health system challenges and stages of NCD care. This framework can guide approaches to fill evidence gaps in this area. Systematic review registration: PROSPERO CRD42014007527.

## Background

The nature of most non-communicable diseases (NCDs) requires a well-integrated healthcare system to meet chronic healthcare needs. This poses a challenge for nations faced with limited human, financial, and infrastructure resources. This is especially true in sub-Saharan Africa (SSA) where NCDs already account for one in four deaths in some countries and the healthcare workforce is deficient by approximately one million workers to meet even basic healthcare needs [1,2]. The proportion of deaths in SSA attributable to NCD risk factors—such as alcohol, hypertension, and household air pollution—has increased in the past two decades [3,4]. In response to these statistics, innovative models of healthcare delivery that integrate novel use of human and technological resources have been regarded as priorities for the SSA region [5].

During the 2011 United Nations High Level Meeting on Non-Communicable Diseases, the use of mobile communication technology in the health arena (mHealth) was highlighted as a key strategy to combat NCDs in low- and middle-income countries (LMICs) [6]. As wireless telecommunications networks have spread rapidly throughout SSA, sending text messages on wireless mobile devices has become a popular means of communication among people in all sectors of society. This rapid growth of mobile phone ownership in LMICs forms the chief rationale for promoting mHealth. There are more mobile phone subscribers in SSA than in the United States or European Union and the cellular market in Africa is expected to grow to 1.12 billion subscribers by 2017 [7].

The evidence supporting the implementation of mHealth in SSA has been focused on HIV/AIDS, malaria, and maternal and child health despite the fact that the burden of NCDs is growing at a faster rate [8-10]. Although the model of the epidemiologic transition postulates that NCDs tend to become dominant causes of death as societies and populations evolve [11], several factors—such as adverse diet trends, mechanization of transport, and increasingly sedentary jobs in LMICs—have led to an acceleration and overlap between the stages of the epidemiologic transition [12,13]. In fact, NCDs such as diabetes mellitus are expected exert an even greater burden in SSA over the next two decades [14]. Our rationale for this review is that despite calls for broad implementation of mHealth in SSA, no systematic literature review has focused on the use of mHealth for NCDs in the region [15]. This review systematically describes the variety of applications of mHealth technologies used in SSA for NCD care; highlights challenges to implementing this type of technology in the region; and, using a framework of health system challenges, describes opportunities for its use with regard to NCD care.

## Methods

### Eligibility criteria

Our search strategy was designed to maximize the likelihood of retrieving relevant citations. Methods of the review were specified in advance and followed published guidance regarding reporting of systematic reviews (i.e., Preferred Reporting Items for Systematic Reviews and Meta-Analyses). Participants included non-hospitalized adults enrolled in both randomized and non-randomized observational studies. Studies were included if the mHealth intervention included unidirectional or bidirectional communication regarding NCDs via mobile phones. Usual care and all comparators were included. Primary outcome measures included behavioral and clinical measures depending on the study design. There was no restriction on the length of follow up.

We included studies published from 1992 through 2012 because short message service (SMS) technology was first used in 1992 [16]. Studies published in English or for which an abstract was available in English were considered. Our search included published manuscripts, abstracts, and grey literature (i.e., blogs, conference proceedings, newspaper articles, technical reports, and working papers).

### Information sources and search

An electronic literature search was conducted using PubMed, EMBASE, Web of Science, Google Scholar, Cochrane Central Register of Controlled Trials, Global Index Medicus, the Association for Computing Machinery Digital Library, CINAHL, IEEE Xplore, PsycINFO, Science Direct, and the Pan African Clinical Trials Registry. We also searched within ClinicalTrials.gov, Knowledge for Health [17], the published proceedings of the American Medical Informatics Association, and within highly cited information and communication technology journals focused on SSA. Grey literature was reviewed similar to peer-reviewed literature. The PubMed search strategy involved a search for studies related to “Sub-Saharan Africa”, “mHealth”, and “Non-communicable diseases”. Full details of our search strategy are in an additional file (see Additional file 1). An analogous search strategy was used for the other databases. References in articles meeting the search criteria were reviewed for additional articles. Letters to the editor and studies whose full-text article were not available were excluded.

### Study selection

Two investigators (GSB, RV) screened records that were retrieved from the search. Each investigator reviewed the titles and abstracts independently to determine whether the study should be included. For conflicting opinions, the records were re-reviewed and if disagreement persisted, a third investigator reviewed the records and the majority opinion was applied.

### Data collection process

A data extraction method was developed based on the Cochrane Consumers and Communication Review Group’s data extraction template. The extraction sheet was pilot-tested on ten randomly selected records and refined. Two investigators (AK, GSB) examined the full-text of the retrieved records and extracted the data. Disagreements were resolved by discussion or by consultation with a third investigator (RV). Information extracted from each study consisted of: characteristics of study participants (including age, sex, disease, and setting of the study), inclusion and exclusion criteria, and outcome measurements. This was a systematic review and no meta-analytic statistical comparisons were performed.

### mHealth Framework for NCDs in SSA

We developed a framework to catalogue the results of the systematic review in order to identify specific areas where evidence to support the efficacy of mHealth interventions in NCD management has been generated as well as those areas where such evidence is lacking. This framework is based on two sets of intersecting parameters: health system challenges and spectrum of disease. The health system challenges to NCD care are prophylaxis and prevention, detection and diagnosis, linkage to care, long-term follow-up, providing high quality care, and coordination of care. The spectrum of disease ranges from those who are healthy to those with complications of disease [18]. The health system challenges manifest differently based on the individual’s disease stage. This framework allows us to visualize the opportunities for mHealth innovations, when used appropriately, to help strengthen health systems and the delivery of essential interventions [9,19]. We recognize that, while not addressed in the framework, other factors such as improvements in built environment, tax and price interventions, as well as advocacy and policy reform are crucial in addressing the NCD burden. This framework draws from existing literature describing the potential of mHealth innovations in SSA, reports on existing mHealth interventions, and the effective interventions listed in the 2005 World Health Organization (WHO) report on Preventing Chronic Diseases [2,19,20].

### Role of the funding source

There was no funding source for this study. The authors had full access to the data and the corresponding author had final responsibility for the decision to submit for publishing. The systematic review registration number (PROSPERO) is CRD42014007527.

## Results

**Figure 1:**
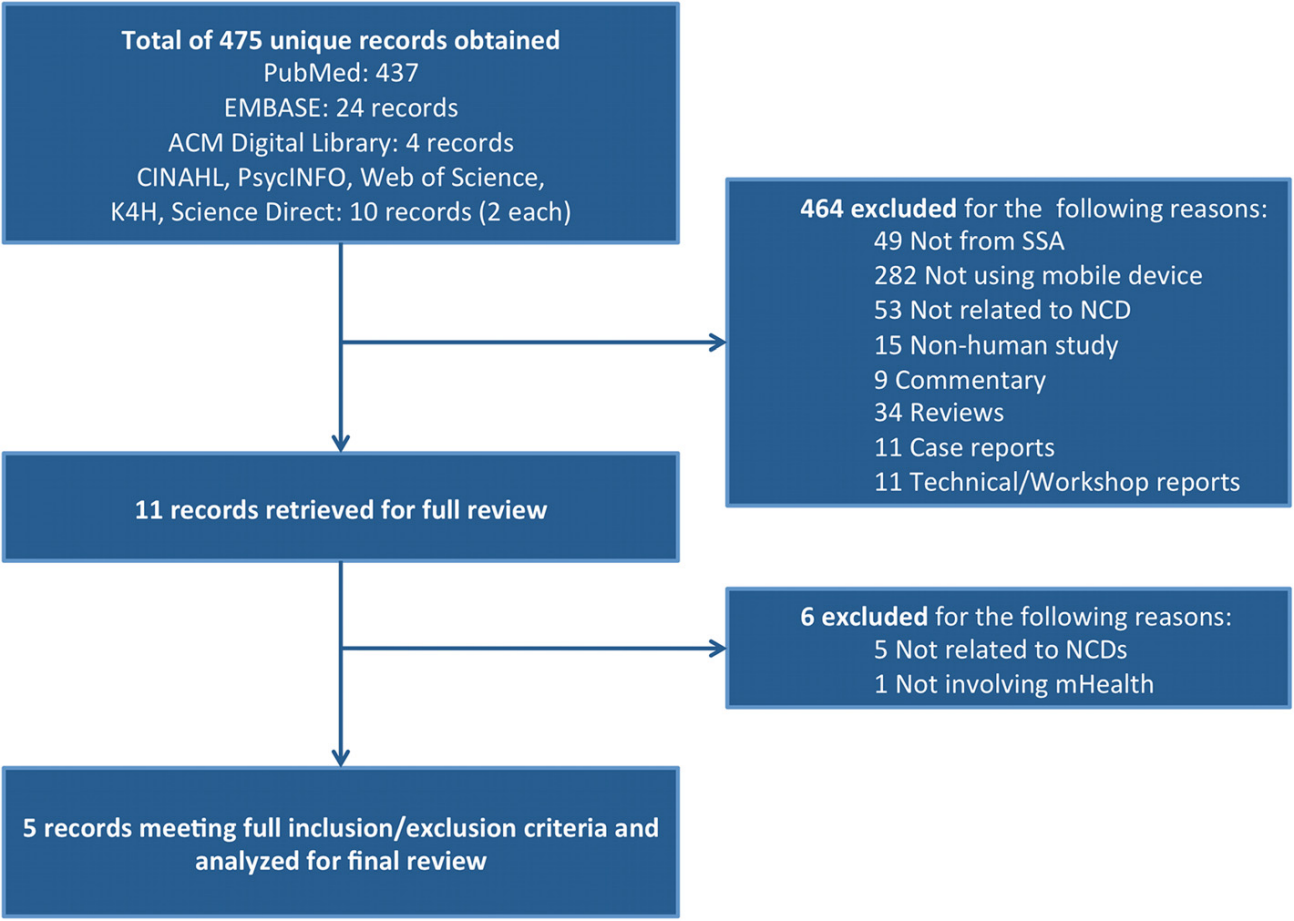
**Reasons for exclusion of studies.** Legend. Schematic diagram of the studies identified by the literature search showing the number of studies excluded for various reasons. Five studies met final inclusion and exclusion criteria.

Our search yielded 475 unique records, of which 464 were excluded for the reasons specified in Figure 1 (see Figure 1). Most of the excluded records were not related to mHealth or were not related to an NCD, as described in an Additional file (see Additional file 2). Eleven manuscripts were reviewed in full. Five of these manuscripts were found to be unrelated to NCDs and one was not an mHealth intervention, resulting in five articles that met inclusion and exclusion criteria. Study characteristics are summarized in Table 1 (see Table 1).

**Table 1:** Characteristics of included studies

**Study (Country)**	**N**	**Participant characteristics**	**Design**	**Outcome measures**	**Findings/Comments**
Chindo et al. [21] (Cameroon)	101	*Age, Sex:* nr	Chart review	Access to mobile phones; Successful contact	Three-fold increase (20% to 60%) in parents with mobile phone from 2007–2010. 59% of parents able to be contacted. 101 guardians of 285 patients.
		*Disease:* Burkitt’s lymphoma			
		*Setting:* Two hospitals			
Pastakia et al. [22] (Kenya)	641	*Age:* 2–88 years	Single center report	Change in HbA1C with home glucose monitoring	Decrease in HbA1C from 13.2% to 10.5% (P < 0.001) after 3–6 months.
		*Sex:* nr			
		*Disease:* Diabetes			
		*Setting:* Teaching hospital and satellite clinics			
Parham et al. [23] (Zambia)	nr	*Age, Sex:* nr	Single center report	Feasibility of digital cervicography intervention	Same-visit cryotherapy; referrals were feasible and bypassed historic barriers to care.
		*Disease:* Cervical cancer			
		*Setting:* Nurses in clinics			
Odigie et al. [24] (Nigeria)	1160	*Age:* 18–82 years	Prospective	Call reason and duration; patient perception; clinic attendance	98% of patients contacted their oncologist. 73% of calls to discuss illness or treatment; 26% to arrange next appointment; 1% of calls to extend social greeting. 100% of intervention patients and 19% of control patients adhered to clinic appointments.
		*Sex:* 57% Female			
		*Disease:* Any cancer			
		*Setting:* Clinics of a teaching hospital			
Rotheram-Borus et al. [25] (South Africa)	22	*Age:* 21–74 years	Single arm intervention	Uptake of text messaging, body mass index, blood pressure, styles of coping, emotional distress, sedentary time	Positive effects on sleep, positive action, and coping. More text messages between buddies than to the program (19 883 vs.1321). 29% response rate to text questions. Higher diastolic blood pressure (+7 mmHg) and less spiritual hope at 6 months. High-texters had higher BMIs and more sedentary time.
		*Sex:* 100% female			
		*Disease:* Diabetes			
		*Setting:* Local clinic			

Abbreviations: *nr* not reported, *hbA1C* glycosylated hemoglobin A1C, *BMI* body mass index.

Chindo et al. investigated the use of mobile phones by guardians of pediatric patients with Burkitt’s lymphoma in two hospitals in Northwest Cameroon [21]. The medical records of 285 patients were reviewed to describe access to mobile phones and whether the guardians could be successfully contacted using the phone number in the medical record. Between 2007 and 2010, access to and ownership of mobile phones increased and most guardians could be contacted (Table 1). The authors conclude that mobile phones are a potentially useful tool for follow-up of patients. However, there were no measured differences in clinical outcomes between those who could and could not be contacted.

A report from Kenya documented the evolution of diabetes care in a resource-constrained setting [22]. As part of a comprehensive program, a mobile phone-based home glucose monitoring program was created in which community health workers provided clinical consultation for diabetic patients via mobile phone. The authors report that patients avoided the need to travel far distances for advice and sought advice sooner than they may have otherwise [22]. In addition, glycosylated hemoglobin levels significantly decreased (Table 1). There was no comparator group and long-term results of the program including clinical outcomes have not been reported.

A study from Zambia reported experiences with a system operated by non-physician health providers in a public sector cervical cancer prevention program [23]. After taking photographs of a suspicious cervical lesion with a mobile phone, nurses in remote settings sent images electronically to an expert consultant for review as well as an SMS message notifying the consultant to review the images. The consultant and nurse were able to communicate via mobile phone while viewing the images simultaneously. There was no formal evaluation of changes in lag time between screening and therapy; however, the authors report that consultation requests were often dealt with while patients were still in the clinic or on the examination table.

A study from a Nigerian teaching hospital investigated mHealth as a tool for improving support during cancer care [24]. Patients were given their oncologists’ mobile phone numbers and advised to call to seek medical advice at any time. The intervention group was compared to a group of cancer patients who were not given their oncologists’ phone number, but who were seen regularly at the clinic. Nearly all patients contacted their oncologists and most calls were to discuss illness or treatment (Table 1). More patients in the intervention arm adhered to clinic appointments than those in the control arm. The study design was such that control patients were simply those that refused to participate in the intervention resulting in non-random assignment. Thus, it is not possible to control for all potential confounders of these results.

The fifth study involved peer support for adult diabetic women via mHealth in South Africa [25]. A series of educational group sessions addressing lifestyle improvements were offered to diabetic women and each was assigned a “text message buddy” to assist with lifestyle changes via SMS. After the sessions, the women were asked health questions via daily text messages. There was no comparator group. The women responded to few of the texted questions, but exchanged a greater number of text messages with each other (Table 1). There were also positive effects on sleep and coping. Women who texted the most also reported the most sedentary time though no causal effect could be established. There was less spiritual hope and higher blood pressure at six months, however, there was no control group with which to compare these changes.

## Discussion

Owing to the growing NCD epidemic and capacity limitations in LMICs, there is great enthusiasm for mHealth innovations in SSA [6]. This comprehensive systematic review of the published and un-published literature offers important insights. We found few studies of variable design examining mHealth in this area. The design of most of the studies did not include comparator arms, were single-center reports, or did not include clinical endpoints. mHealth for NCDs appears feasible for follow-up of patients, can be integrated into a larger NCD program, and facilitates peer support networks in SSA. Research in this area has been limited but must be pursued in order to justify widescale implementation of mHealth strategies that are also effective in reducing the escalating burden of NCDs in SSA.

There have been a number of systematic reviews of the effectiveness of mHealth for improving adherence to clinic attendance [26], improving health care service delivery [27], and healthcare in general [28]. None of these reviews included studies from SSA, even when focused on developing countries, and few of them relate to NCDs [29]. Reviews that have included such countries have usually focused on HIV/AIDS or family planning/pregnancy [8]. This systematic review was specifically focused on mHealth applied to NCDs in SSA and included an extensive search of the grey literature. We agree with Tomlinson et al., that despite the calls to scale up mHealth interventions in SSA, there are few current mHealth interventions that meet standards for scale up [15], especially for NCDs. Our review advances the state of knowledge in this field owing to the inclusion of studies from SSA and our focus on NCDs. The fact that mHealth has not been widely investigated in SSA for NCDs may relate to low awareness of these conditions in comparison to communicable diseases and exemplifies a disparity in the types of research in this field [30].

### An mHealth framework for non-communicable diseases in sub-Saharan Africa

**Figure 2:**
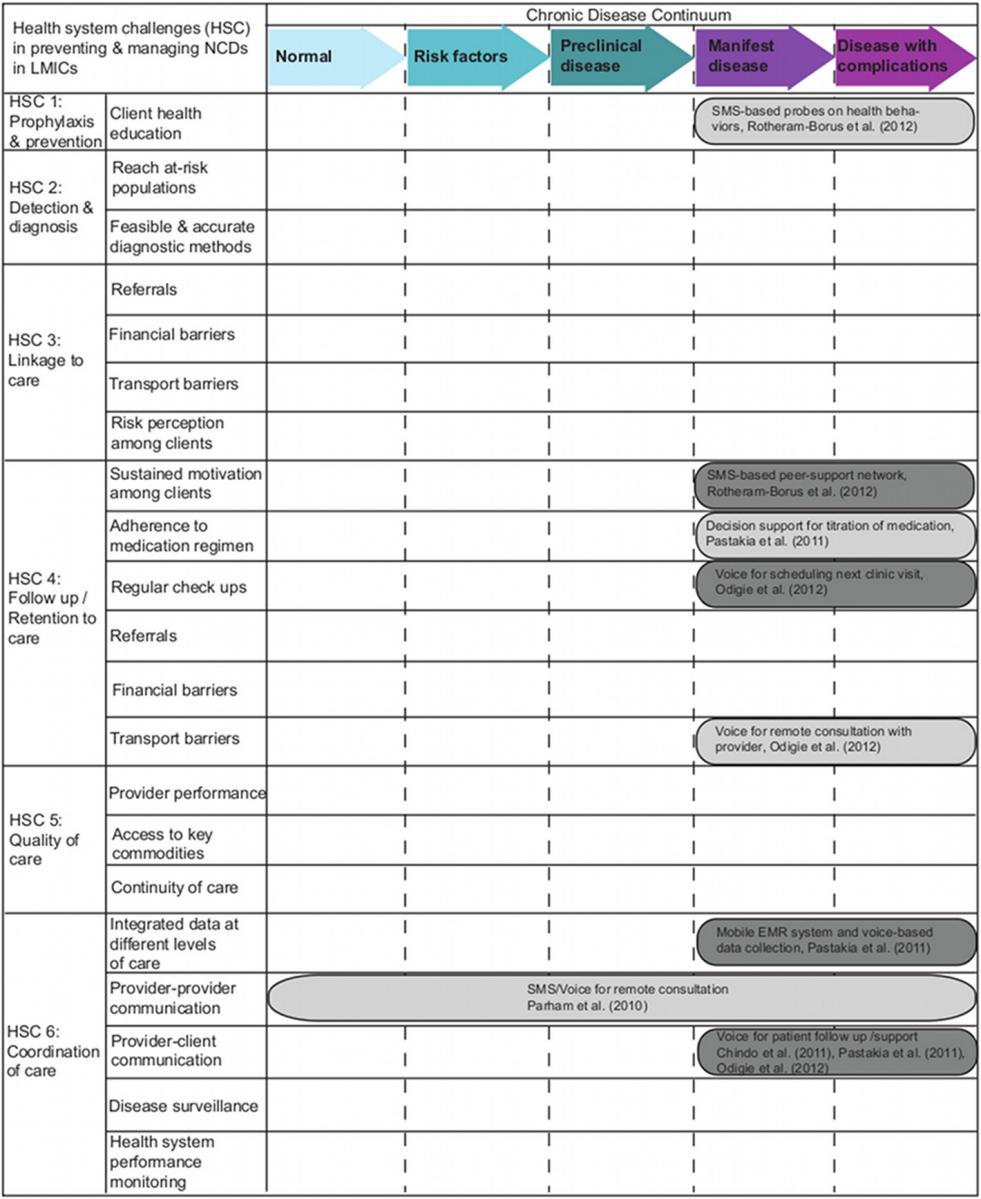
**Results of the systematic review embedded in the mHealth strategies framework to address health systems challenges to non-communicable disease care.** Legend. The chronic disease continuum includes healthy people with no apparent disease through patients with complications of non-communicable diseases (NCDs). Health System Challenges (HSC) one through six are shown alongside examples of specific barriers as they relate to NCDs. The mHealth programs found in the literature search are displayed where these programs intersect HSCs at points along the chronic disease continuum. There remain many opportunities for implementation of mHealth for non-communicable diseases (NCDs) in sub-Saharan Africa and other LMIC regions broadly.

Due to the scant amount of evidence, we conclude that there are insufficient data to support the notion that mHealth as applied to NCDs in SSA is effective and that a more comprehensive, systematic approach to knowledge generation is required. We provide a framework depicted in Figure 2 that incorporates the health system challenges inherent to NCDs and the continuum of NCD development to identify the optimal mHealth strategies for SSA and potentially for LMICs broadly. Efforts to catalog and synthesize evidence for mHealth for NCDs in SSA along such lines may assist stakeholders and policy makers in evaluating innovations and strategies that merit additional further investment. For each of the health system challenges, we provide examples of mHealth implementations when they exist. Although not every category has an existing implementation related to NCDs, it is important to note that there may be many NCD mHealth implementations that have been deployed but that have not been tested and/or published during the period of this review. The era of so-called “pilotitis” of mHealth projects and unfettered proliferation of mHealth strategies is well recognized [31,32] and there is dire need to evaluate for health impact alongside implementation. For NCDs, the need for high-quality evidence to inform many important decisions regarding diagnosis, prevention and treatment is too great for research and implementation to operate independently [33]. This highlights the need to incorporate implementation research, monitoring and evaluation as part of mHealth deployments in this field and region in a systematic manner (Figure 2).

#### *Health system challenge 1: prophylaxis and prevention*

Poor knowledge of preventive measures is a key contributor to the rising incidence of NCDs in SSA [34]. Mobile phones have been leveraged for client health education using text messaging to address risk factors for NCDs in developed countries [35]. In SSA, mHealth for prevention or prophylaxis has been implemented for reproductive, maternal, newborn, and child health (e.g., SMS-based gestational age-appropriate health messages for pregnant women in South Africa [36]; Chipatala Cha Pa Foni hotline service in Malawi [37]; and mobile for reproductive health in Tanzania [38]). We found no studies that used mHealth for prophylaxis or prevention of NCDs in patients without manifest disease (Figure 2).

#### *Health system challenge 2: detection and diagnosis*

Decision support is an attractive feature of mHealth whereby healthcare personnel can be provided with automated algorithms that assist in diagnosis and protocol adherence [39]. This has been demonstrated in developed countries and interventions with decision support for hypertension, diabetes, and related NCDs are underway in SSA (e.g., D-tree International) [40,41]. The integration of biosensors and powerful cameras with mobile phones offers opportunities for screening of NCDs. Transmission of reports, images, or queries can allow frontline health workers to connect with trained providers remotely and thus, improve the quality of task shifting. This approach has a longer track record in dermatopathology and communicable diseases though adoption for NCDs has been demonstrated in Zambia [23,42].

#### *Health system challenge 3: linkage to care*

Low awareness of a condition is a major limiting factor linking patients to care for NCDs in LMICs. mHealth may increase awareness through education and behavior change communication. For example, Project Masiluleke in South Africa [43] sends approximately one million text messages with health information on HIV/AIDS and tuberculosis to users daily. Such systems can be tailored to provide information regarding NCDs as has been done in developed countries [27].

Inadequate infrastructure and high transportation costs pose great challenges to physically linking a patient with a provider in LMICs [44]. Linking individuals to transportation services via messaging, voice, or global positioning system technology may increase a patient’s likelihood of linkage. Similarly, mHealth applications in the banking sector (e.g., Safaricom’s M-Pesa) allow customers to hold and transfer cash balances on their subscriber identity module (SIM) cards. Many retail shops and grocery stores accept these forms of payments, as could clinics, hospitals, and pharmacies. We found no published studies that used mHealth for linkage to care for NCDs in SSA, but more insights may be soon forthcoming [45].

#### *Health system challenge 4: follow up/retention to care*

The mHealth strategies with an evidence base to improve retention often deal with SMS reminders about medication and appointment adherence [26,46]. mHealth can also support retention to care by assisting patients with financial barriers to care, securing transportation, and assist providers with a referral and consultation base (e.g., Changamka’s smart-card based insurance services [47]). Financial and banking applications may have a particularly receptive niche in the NCD field since care for these diseases are not usually subsidized.

Reminder systems can play a role in improving retention to care. One strategy is pill bottle-based reminder systems to increase adherence to medication regimens (e.g., SIMpill [48]). Other forms of reminders include those about upcoming medical appointments, alerts for daily monitoring (e.g., of blood glucose or pressure levels), and motivational reminders for behavior change (e.g., promoting daily exercise) as demonstrated in South Africa [25].

#### *Health system challenge 5: quality of care*

mHealth can support provider education, training, work planning, decision support, reminders, and supportive supervision to improve quality of care. Mobile phone-based applications such as the electronic Mobile Open-source Comprehensive Health Application [49] allow providers to access training videos and could improve workforce skill level. mHealth can also aid in supply maintenance of key commodities between various sites. Supply chain management systems such as the public-private partnership SMSforLife uses electronic mapping technology and SMS to prevent stock-outs of essential medications [50]. We found no published studies of this application of mHealth in SSA for NCDs.

#### *Health system challenge 6: coordination of care*

The challenges for coordination of care relate to high quality provider-patient interactions, provider-provider interactions, integrated data at different levels of care, and disease surveillance. While there is evidence from developed countries that mHealth improves NCD control, the outcomes of mHealth to coordinate care in SSA have been mixed [51]. The South African SMS-based program for diabetic women, for example, showed that participants sent more messages to each other than respond to text questions from the program. Despite this, there was some benefit seen in sleeping and coping [25].

Provider-provider communication via closed user groups provides a reliable communication system for consultations and referrals. An example is the Mobile Doctors Network/Medicareline program (MDNet) in Ghana that provides free voice and text services to all registered physicians and has led to beneficial impact on overall patient care [9].

Integrating data at different levels of care can be facilitated by mHealth via electronic health records. Using smartphones in western Kenya, for example, investigators recorded clinical data from over 18 000 patients over a six-month period during home visits for HIV testing [52]. The use of such systems could ensure continuity of care and informed decision making in the case of NCDs as well.

## Conclusions

In summary, while there is great enthusiasm for the application of mHealth for NCDs in SSA, implementation is limited and evidence for a positive effect on health is sparse. After an extensive search of the published and “grey” literature, our systematic review yielded only five reports using mHealth in this field. To address this gap between promise and evidence, we have presented a framework highlighting mHealth strategies across the continuum of care for NCDs stratified by health systems challenges in SSA. There are numerous opportunities for research and implementation of mHealth in these domains that have not been pursued. Based on our findings, we urge mHealth developers, users and scientists to work collaboratively to fill in the many gaps we have displayed in Figure 2. In addition, the literature lacks controlled trials or other study designs with comparator arms and we found few mHealth deployments that incorporate monitoring or evaluation of effectiveness. Moreover, the causal pathways that link mHealth to improved NCD health warrant further investigation. Our framework outlines health system challenges that may be intermediate outcomes to reflect possible causal pathways between mHealth application use and impact on health outcomes of interest. If the promise of mHealth to transform care in the region is to be realized, focused implementation research studies building on the framework provided must be pursued for those with NCDs as well as the larger population at risk for NCDs.

## Abbreviations

LMIC: Low- and middle-income country; mHealth: mobile health; NCD: Non-communicable disease; SIM: Subscriber identity module; SMS: Short message service; SSA: Sub-Saharan Africa; WHO: World Health Organization.

## Competing interests

All authors state that no conflicts of interest exist.

## Authors’ contributions

GSB conceived the study, refined the study design, performed the literature search, screened records, reviewed records retrieved from the search, extracted data, reviewed data from each study, and wrote the first draft of the manuscript. RV refined the study design, screened records, extracted data, reviewed records retrieved from the search, and reviewed data from each study. LV designed the framework figure. AK performed the literature search, extracted data, and reviewed data from each study. All authors contributed to data interpretation, data interpretation, and writing. All authors reviewed the manuscript, provided important intellectual input, and gave final approval for the final version to be submitted.

## Authors’ information

LV is a member of the World Health Organization mHealth Technical Evidence Review Group (WHO mTERG) and a consultant for the Department of Reproductive Health and Research of WHO. MW is co-chair of the mHealth Alliance Evidence Working Group and is a member of the WHO mTERG.

## Supplementary Material

Additional file 1 **PubMed Search Strategy.** Details of the search criteria used for the PubMed search. Click here for file

Additional file 2 **Reasons for Exclusion.** List of all studies retrieved by the search and reasons for inclusion/exclusion from the analysis. Click here for file
