# Supplementary material for: Mobile health for non-communicable diseases in Sub-Saharan Africa: a systematic review of the literature and strategic framework for research
Source: Global Health. 2014 Jun 13;10:49. doi: 10.1186/1744-8603-10-49 (PMC4064106; doi:10.1186/1744-8603-10-49)
Supplement: Additional file 1 — PubMed Search Strategy. Details of the search criteria used for the PubMed search. [file 1744-8603-10-49-S1.pdf]

Additional File 1

PubMed search strategy

(1) Africa South of the Sahara (Mesh) or sub-sahara* (truncated textword) or africa (textword); AND

(2) “personal digital assistant” (textword), sms (textword), short message servic* (truncated textword), text messag* (truncated textword), mobile phone* (truncated textword), cell phone* (truncated textword), Computers, Handheld (Mesh), Cellular Phone (MeSH), Telephone (Mesh), telecommunicat* (truncated textword), wireless (textword), or telemedicine (textword); AND

(3) chronic disease (Mesh), “chronic disease” (textword), non-communicable (textword), noncommunicable (textword) or NCD* (truncated textword)
